# Nationwide sample data analysis of additional surgery rate after anterior or posterior cervical spinal surgery

**DOI:** 10.1038/s41598-023-33588-z

**Published:** 2023-04-18

**Authors:** Woon Tak Yuh, Minjung Kim, Yunhee Choi, Junghoon Han, Junhoe Kim, Taeshin Kim, Chun Kee Chung, Chang-Hyun Lee, Sung Bae Park, Kyoung-Tae Kim, John M. Rhee, Moon Soo Park, Chi Heon Kim

**Affiliations:** 1 Department of Neurosurgery, Hallym University Dongtan Sacred Heart Hospital, 7 Keunjaebong-gil, Hwaseong-si, Gyeonggi-do 18450 Republic of Korea; 2 Department of Neurosurgery, Seoul National University Hospital, 101 Daehak-ro, Jongno-gu, Seoul, 03080 Republic of Korea; 3 Division of Medical Statistics, Medical Research Collaborating Center, Seoul National University Hospital, 101 Daehak-ro, Jongno-gu, Seoul, 03080 Republic of Korea; 4 Department of Neurosurgery, Seoul National University College of Medicine, 103 Daehak-ro, Jongno-gu, Seoul, 03080 Republic of Korea; 5 Department of Brain and Cognitive Sciences, College of Natural Science, Seoul National University, 1 Gwanak-ro, Gwanak-gu, Seoul, 08826 Republic of Korea; 6 Department of Neurosurgery, Seoul National University Boramae Hospital, Boramae Medical Center, 20 Boramae-ro 5-gil, Dongjak-gu, Seoul, 07061 Republic of Korea; 7 Department of Neurosurgery, Kyungpook National University Hospital, 130 Dongdeok-ro, Jung-gu, Daegu, 41944 Republic of Korea; 8 Department of Neurosurgery, School of Medicine, Kyungpook National University, 680 Gukchaebosang-ro, Jung-gu, Daegu, 41944 Republic of Korea; 9 Department of Orthopaedic Surgery, Emory University School of Medicine, Atlanta, GA 30322 USA; 10 Department of Orthopedics, Hallym University Dongtan Sacred Heart Hospital, 22 Gwanpyeong-ro 170 Beon-gil, Dongan-gu, Anyang-si, Gyeonggi-do 14068 Republic of Korea; 11 Department of Medical Device Development, Seoul National University College of Medicine, 103 Daehak-ro, Jongno-gu, Seoul, 03080 Republic of Korea

**Keywords:** Epidemiology, Outcomes research, Diseases

## Abstract

Surgical outcomes of degenerative cervical spinal disease are dependent on the selection of surgical techniques. Although a standardized decision cannot be made in an actual clinical setting, continued education is provided to standardize the medical practice among surgeons. Therefore, it is necessary to supervise and regularly update overall surgical outcomes. This study aimed to compare the rate of additional surgery between anterior and posterior surgeries for degenerative cervical spinal disease using the National Health Insurance Service-National Sample Cohort (NHIS-NSC) nationwide patient database. The NHIS-NSC is a population-based cohort with about a million participants. This retrospective cohort study included 741 adult patients (> 18 years) who underwent their first cervical spinal surgery for degenerative cervical spinal disease. The median follow-up period was 7.3 years. An event was defined as the registration of any type of cervical spinal surgery during the follow-up period. Event-free survival analysis was used for outcome analysis, and the following factors were used as covariates for adjustment: location of disease, sex, age, type of insurance, disability, type of hospital, Charles comorbidity Index, and osteoporosis. Anterior cervical surgery was selected for 75.0% of the patients, and posterior cervical surgery for the remaining 25.0%. Cervical radiculopathy due to foraminal stenosis, hard disc, or soft disc was the primary diagnosis in 78.0% of the patients, and central spinal stenosis was the primary diagnosis in 22.0% of them. Additional surgery was performed for 5.0% of the patients after anterior cervical surgery and 6.5% of the patients after posterior cervical surgery (adjusted subhazard ratio, 0.83; 95% confidence interval, 0.40–1.74). The rates of additional surgery were not different between anterior and posterior cervical surgeries. The results would be helpful in evaluating current practice as a whole and adjusting the health insurance policy.

## Introduction

The surgical outcome of degenerative cervical spinal disease is largely dependent on the selection of surgical techniques^1–4^. Several studies, including randomized controlled trials, cohort studies, and systematic reviews, have highlighted the benefits of each surgical technique for degenerative cervical spinal disease^1,2,5–24^. Generally, cervical surgery techniques are largely divided into anterior and posterior cervical surgeries^1,6–8,10,12^. The anterior cervical surgery is recommended when the compression lesion is located at less than three levels. For such cases, anterior cervical discectomy and fusion (ACDF) or anterior cervical corpectomy and fusion are the representative surgical techniques^5,14,19^. When the compressive lesions span more than three levels, posterior cervical surgeries such as laminoplasty or laminectomy with or without instrumented fusion are recommended^19^. For kyphotic cervical spine, anterior cervical surgery or posterior laminectomy with instrumentation is recommended^4,20,22,25–27^. Although previous studies have shown similar clinical outcomes between anterior and posterior cervical surgeries, the anterior surgery showed better cervical alignment^1,5–17^. However, the incidence of surgical complications seemed to be higher for anterior cervical surgeries, except for C5 palsy^1–3,5–18,20,21,23,24,28–31^. Therefore, selecting the correct cervical surgical technique is not a straightforward decision, and surgeons should refer to previous studies and discuss their recommendations with patients for shared decision-making. Although the relevant cervical surgical technique for an actual clinical setting is yet to be unanimously established, continuous education is being conducted through research publications and academic conferences to standardize the medical practice globally.

Therefore, it is crucial to supervise the overall outcomes of cervical surgeries and update them regularly^21^. Although prospective studies or systematic analyses could provide robust evidence regarding a surgical guideline, the analyses of nationwide registered patient data would be an appropriate way to supervise the overall outcomes of cervical surgeries^1,2,32,33^. In the Republic of Korea (ROK), all citizens are beneficiaries of the national health insurance service (NHIS) for more than 20 years. The nationwide inpatient and outpatient data on diseases and services (i.e., procedures and operations) are coded and registered in the National Health Insurance Corporation and the Health Insurance Review & Assessment Service (HIRA) databases^34–39^. This study aimed to compare the rate of additional surgery after anterior and posterior cervical surgeries for degenerative cervical spinal disease using the nationwide database.

## Methods

### Data source

This study used the patient data from the National Health Insurance Service-National Sample Cohort (NHIS-NSC). The NHIS-NSC is a representative sample cohort, and 1,000,000 people (2.1% of the total Korean population) were randomly selected from a total population of 48,438,292 in 2006 (https://nhiss.nhis.or.kr/bd/ab/bdaba021eng.do)^32,33,39^. The nationwide database of NHIS was developed to record personal information, demographics, and medical treatment data of all patients for health insurance purpose^32,33,39,40^. The disease codes were standardized based on the 10th version of the International Classification of Diseases (ICD-10), and the procedure codes were standardized for medical fee claims^32,34,36,41^. In this study, NHIS-NSC data up to 2015 was selected for analysis, while maintaining representativeness and protection of personal information (NHIS-3017-2-494)^32,33,39^. Systematic stratified random sampling with proportional allocation within each stratum was conducted^39^. The included strata comprised sex, age, location, and health insurance type (insured employees, insured self-employed individuals, or medical aid beneficiaries)^32,33,39^. The resident registration number was replaced with a newly assigned eight-digit personal ID that enabled longitudinal follow-ups for all patients up to 2015^39^. During the follow-up period, the cohort was annually updated, and a representative sample of newborns was included to maintain the size of the cohort^32,33,39^. The cohort included claims from hospitals, pharmacies, and oriental medicine clinics. Each patient record in the NHIS-NSC can be traced back to 2002 and followed-up to 2015^32,33^.

### Patients

**Figure 1:**
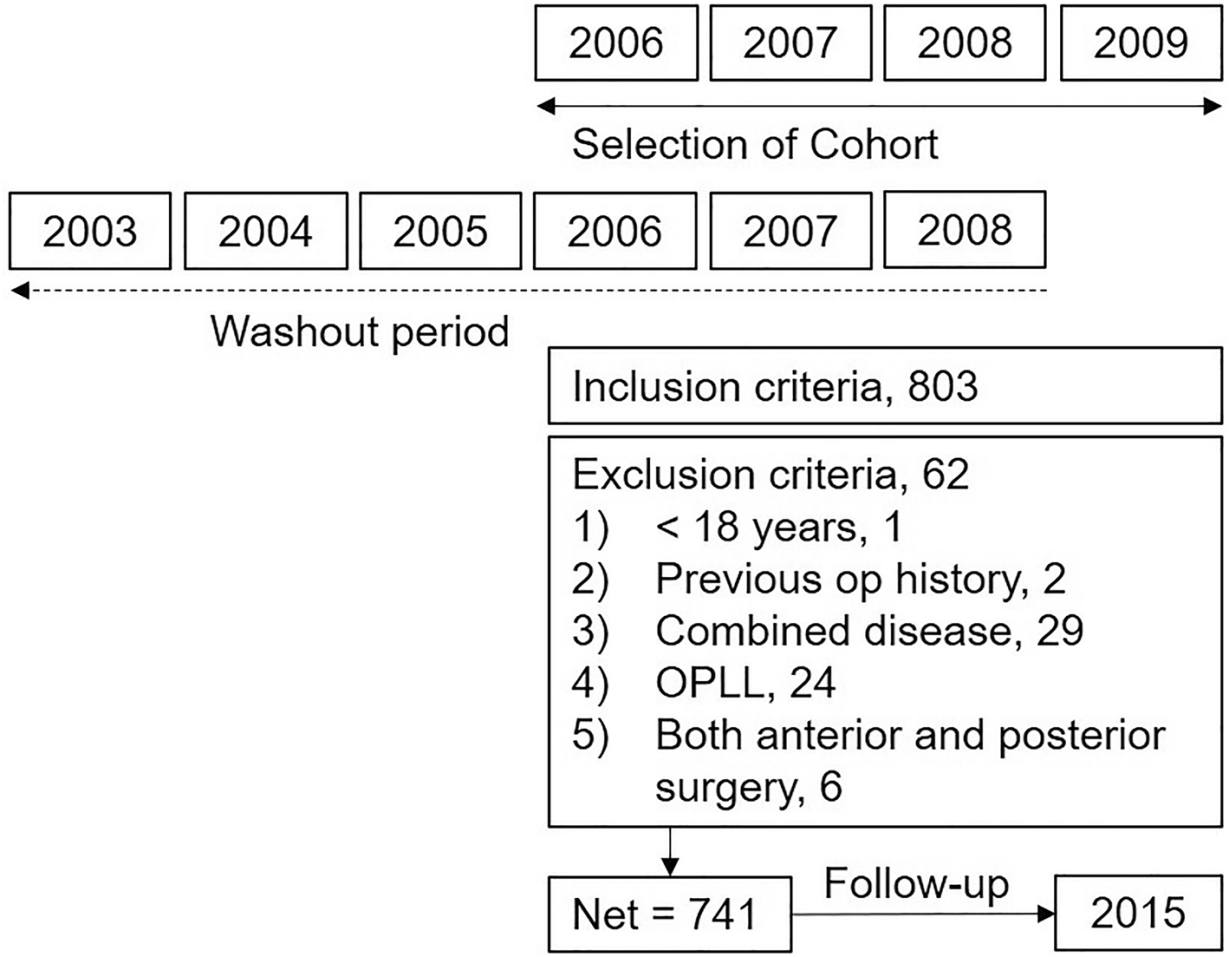
Flow diagram of the included patients. Patients were selected from 2006 to 2009, and the washout period was 3 years for each year of selection. Finally, 741 patients were selected and followed-up until 2015.

This retrospective cohort study included adult patients, over the age of 18 years, with degenerative cervical spinal disease, such as cervical spinal stenosis or cervical spinal disc herniation. The inclusion criteria were as follows: (1) patients who had cervical spinal surgery from 2006 to 2009 with disease codes (cervical spinal central stenosis, cervical myelopathy, M9931, M9941, M9951, M47-, M480-, M500-, M995-, M532-, M4892, M4893; cervical spinal foraminal stenosis, M9961, M9971; cervical disc herniation, M501-, M502-, M4722, M503-, M508-, M509-, M541-) as either primary or secondary diagnosis in registered claim data for surgery and (2) patients with procedure codes of cervical spinal surgery (cervical anterior discectomy/corpectomy and fusion, N0451, N0464, N2463; cervical posterior laminectomy, N1497, N2497; cervical posterior discectomy, N0491, N1491; cervical posterior laminoplasty, N2491, N2492; cervical posterior decompression and fusion, N0467, N2469). When the patients had multiple diseases, the representative disease code followed the hierarchical coding algorithm proposed by Martin et al., namely cervical spinal canal stenosis, cervical spinal foraminal stenosis, and cervical spinal disc herniation^42^. Based on the above inclusion criteria, 803 patients were selected for this study (Fig. 1). The exclusion criteria were as follows: (1) age less than 18 years (n = 1); (2) history of cervical spinal surgery in the past 3 years (washout period, n = 2); (3) combined diseases such as spinal fracture, pathologic fracture, inflammatory joint disease, and cancer (n = 29); (4) ossification of the posterior longitudinal ligament (n = 24); and (5) both anterior and posterior cervical surgeries (n = 6). Therefore, data from 741 patients were finally included in the analysis. Each patient was followed-up until 2015 using their unique ID, and the follow-up period was a minimum of 6 and a maximum of 9 years. The use of medical services, including outpatient clinics and admissions, were recorded in the database. The requirement for informed consent was waived because the data were de-identified. All methods were performed in accordance with the relevant guidelines and regulation, and the Institutional Review Board of the Seoul National University Hospital approved the review and analysis of the data (No. 2012-014-1177).

### NHIS in the ROK^41^

The ROK adopted a government-controlled NHIS, which was funded by taxpaying citizens. The NHIS is a service provider, and the HIRA controls approvals for claims reimbursement. In the ROK, healthcare centers are categorized into four types by law: clinics, hospitals, general hospitals, and tertiary-referral hospitals^41^. The general hospitals have more than 99 beds and at least seven departments among internal medicine, general surgery, obstetrics and gynecology, pediatrics, diagnostic radiology, anesthesiology, pathology, and laboratory medicine, with at least one board-certified doctor in each department^36^. The tertiary-referral hospitals are designated from among the general hospitals by the government. The tertiary-referral hospitals should have at least 20 departments and include the basic requirements of a general hospital along with a residency training program, at least five operating rooms, and a variety of imaging/diagnostic tools used for computed tomography, magnetic resonance imaging, electromyography, angiography, gamma camera radiography, and Holter cardiac monitoring. In addition, the proportion of patients with complicated diseases (as designated by the Ministry of Health and Welfare) should be more than 12% of the total number of annual inpatients^36,41^. A hospital is defined as a healthcare center lacking any of the essential departments of the general hospital or having between 30 and 99 beds. The clinics have fewer than 30 beds^36^. The NHIS allows individuals to choose their medical service providers. The deduction rate is 50% for outpatient clinics and 20% for admissions (https://hineca.kr/1913). A “fee for service” is the traditional reimbursement system, and hospitals request the reimbursement after discharge of patients. Reimbursements are approved by the board of the HIRA. For reimbursement, the disease codes are standardized according to the ICD-10. The codes of medical services are standardized by the NHIC and HIRA, and the medical fee claims are filed with the NHIC. Detailed surgical and nonsurgical management is determined by attending physicians. However, they are required to follow the guidelines of the national health insurance for reimbursements, such as prescribing a 3 months conservative treatment before surgery for radiologically confirmed central/ foraminal stenosis or myelopathy^34,36,41^. Surgery for mild stenosis is usually not accepted for reimbursement.

### Statistical analysis

This study aimed to longitudinally compare the cumulative rate of additional surgery after anterior and posterior cervical surgeries. The patient characteristics are summarized as mean ± standard deviation for continuous variables and as frequencies (proportions) for categorical variables. The additional surgery event was defined as the registration of the patient for a cervical spinal surgery during the follow-up period. This included both revision surgery at the index level and surgery outside of the index level, because the database cannot differentiate between them. Censoring was performed when the patient reached the final follow-up period without a cervical spinal surgery or upon death. To compare between the cumulative incidences of additional surgeries after anterior and posterior cervical surgeries, the adjusted subhazard ratio (SHR) was calculated using Fine and Gray competing risk regression analysis. The adjusted factors included location of the disease (central vs. foraminal), sex, age (continuous), type of insurance (general vs. Medicare), disability (no, mild, or severe), type of hospital (tertiary-referral hospital, general hospital, hospital, or clinic), Charles comorbidity index (CCI; no vs more than 1), diabetes mellitus (no vs yes), diabetes (no vs yes), and osteoporosis (no vs yes). Comorbidity was defined as more than two visits to outpatient clinics or hospital admission for more than 2 days with disease codes during the preceding 1 year^33,43^. CCI was assessed based on comorbidities^33,44^. The severity of physical disability was categorized as none, mild, or severe^33,45^. The degree of disability was evaluated based on the national severity index ranging from 1 (severe) to 6 (mild). Patients with an index of 1–2 were considered severe (ambulation was dependent on aids or a wheelchair or the patient was bedridden) and those with 3–6 were classified as mild (independent ambulation without aids)^45^. The hospitals were categorized as tertiary-referral hospitals (≥ 300 beds), general hospital (100–300 beds), hospitals (30–99 beds), and clinics (30 beds) based on their size and capacity^33,35,36^. The proportionality of the hazard ratio was checked using restricted LML plot and interaction terms with time. All analyses were conducted using SAS statistical software version 9.4 (SAS Institute Inc., Cary, NC, USA), and *P* < 0.05 (two-tailed) indicated statistical significance.

## Results

Table 1 summarizes the characteristics of 741 patients included in this study. Anterior cervical surgery was performed in 75% (n = 556) and posterior cervical surgery was performed in 25.0% (n = 185) of the patients. Cervical spinal central stenosis was the primary diagnosis in 22% (n = 163) and cervical foraminal radiculopathy due to foraminal stenosis, hard disc, or soft disc was the primary diagnosis in 78% (n = 578) of the patients. Among the 556 patients who underwent anterior cervical surgery, 19.1% (n = 106) were diagnosed with central stenosis and 80.9% (n = 450) were diagnosed with foraminal radiculopathy. Among the 185 patients who underwent posterior cervical surgery, 30.8% (n = 57) had central stenosis and 69.19% (n = 128) had foraminal radiculopathy (*P* < 0.01). The mean age of the patients was 51.8 ± 11.0 years, and 62.9% of the patients were in their 40 s or 50 s. Most patients (96%) were beneficiaries of the general national health insurance. Comorbidities were combined in 62.5% of the patients, and common comorbidities were mild liver disease (16.3%), peptic ulcer disease (12.3%), and diabetes without complications (11.7%). Osteoporosis was present in 18.5% of the patients. During the follow-up period (median; 7.3 years), additional surgery was performed on 6.5% (12 of 185) patients after posterior cervical surgery and 5.0% (28 of 556) patients after anterior cervical surgery.

**Table 1:** Characteristics of the patients.

	Total	Posterior surgery	Anterior surgery	*p* value
	n = 741	n = 185	n = 556	
Surgical method, n (%)				
Posterior cervical surgery	185 (24.97)			
Decompression and fusion	20			
Laminectomy	41			
Laminoplasty	22			
Discectomy	102			
Anterior cervical surgery	556 (75.03)			
ACCF	18			
ACDF	538			
Disease, n (%)				
Central stenosis	163 (22)	57 (30.81)	106 (19.06)	0.0008^a^
Foraminal stenosis/disc herniation	578 (78)	128 (69.19)	450 (80.94)	
Sex, n (%)				
Male	476 (64.24)	119 (64.32)	357 (64.21)	0.9773^a^
Female	265 (35.76)	66 (35.68)	199 (35.79)	
Age, years (%)				
Mean ± SD	51.77 ± 10.95	52.77 ± 11.42	51.44 ± 10.78	0.1517^b^
Median [Min, Max]	51 [20, 87]	51 [20, 87]	51[22, 86]	
18–39	86 (11.61)	17 (9.19)	69 (12.41)	0.0425^a^
40–59	466 (62.89)	117 (63.24)	349 (62.77)	
60–69	149 (20.11)	34 (18.38)	115 (20.68)	
70 and above	40 (5.40)	17 (9.19)	23 (4.14)	
Insurance, n (%)				
General	711 (95.95)	174 (94.05)	537 (96.58)	0.1306^a^
Medicare	30 (4.05)	11 (5.95)	19 (3.42)	
Disability, n (%)				
None	665 (89.74)	155 (83.78)	510 (91.73)	0.0050^a^
Mild	62 (8.37)	26 (14.05)	36 (6.47)	
Moderate	14 (1.89)	4 (2.16)	10 (1.80)	
Type of hospital, n (%)				
Tertiary-referral hospital	209 (28.21)	52 (28.11)	157 (28.24)	0.0432^a^
General hospital	194 (26.18)	55 (29.73)	139 (25.00)	
Hospital	313 (42.24)	67 (36.22)	246 (44.24)	
Clinic	25 (3.37)	11 (5.95)	14 (2.52)	
Charles comorbidity index, n (%)				
Mean ± SD	0.72 ± 1.1	0.8 ± 1.12	0.7 ± 1.1	0.0976^c^
Median [Min, Max]	0 [0, 6]	0 [0, 6]	0 [0, 5]	
0	463	103	360	
1	105	39	66	
2	116	28	88	
3	33	9	24	
4	21	5	16	
5	2	0	2	
6	1	1	0	
0	463 (62.48)	103 (55.68)	360 (64.75)	0.0273^a^
≥ 1	278 (37.52)	82 (44.32)	196 (35.25)	
(1) Myocardial infarction	38 (5.13)	12 (6.49)	26 (4.68)	
(2) Congestive heart failure	6 (0.81)	1 (0.54)	5 (0.90)	
(3) Peripheral vascular disease	61 (8.23)	13 (7.03)	48 (8.63)	
(4) Cerebrovascular disease	20 (2.70)	5 (2.70)	15 (2.70)	
(5) Dementia	0 (0)	0 (0)	0 (0)	
(6) Chronic pulmonary disease	79 (10.66)	20 (10.81)	59 (10.61)	
(7) Rheumatic disease	40 (5.40)	16 (8.65)	24 (4.32)	
(8) Peptic ulcer disease	91 (12.28)	28 (15.14)	63 (11.33)	
(9) Mild liver disease	121 (16.33)	28 (15.14)	93 (16.73)	
(10) DM with complication	72 (9.72)	22 (11.89)	50 (8.99)	
(11) DM without complication	87 (11.74)	27 (14.59)	60 (10.79)	
(12) Hemiplegia or paraplegia	26 (3.51)	11 (5.95)	15 (2.70)	
(13) Renal disease	3 (0.40)	0 (0)	3 (0.54)	
(14) Any malignancy, including lymphoma and leukemia	16 (2.16)	5 (2.70)	11 (1.98)	
(15) Moderate or severe liver disease	1 (0.13)	0 (0)	1 (0.18)	
(16) Metastatic solid tumor	0 (0)	0 (0)	0 (0)	
(17) AIDS	0 (0)	0 (0)	0 (0)	
Diabetes mellitus, n (%)				
No	618 (83.40)	147 (79.46)	471 (84.71)	0.0962^a^
Yes	123 (16.60)	38 (20.54)	85 (15.29)	
Osteoporosis, n (%)				
No	604 (81.51)	148 (80)	456 (82.01)	0.5410^a^
Yes	137 (18.49)	37 (20)	100 (17.99)	

*SD* standard deviation, *DM* diabetes mellitus, *AIDS* acquired immunodeficiency syndrome.

^a^Chi square test.

^b^T-test.

^c^Wicoxon Rank Sum test.

**Figure 2:**
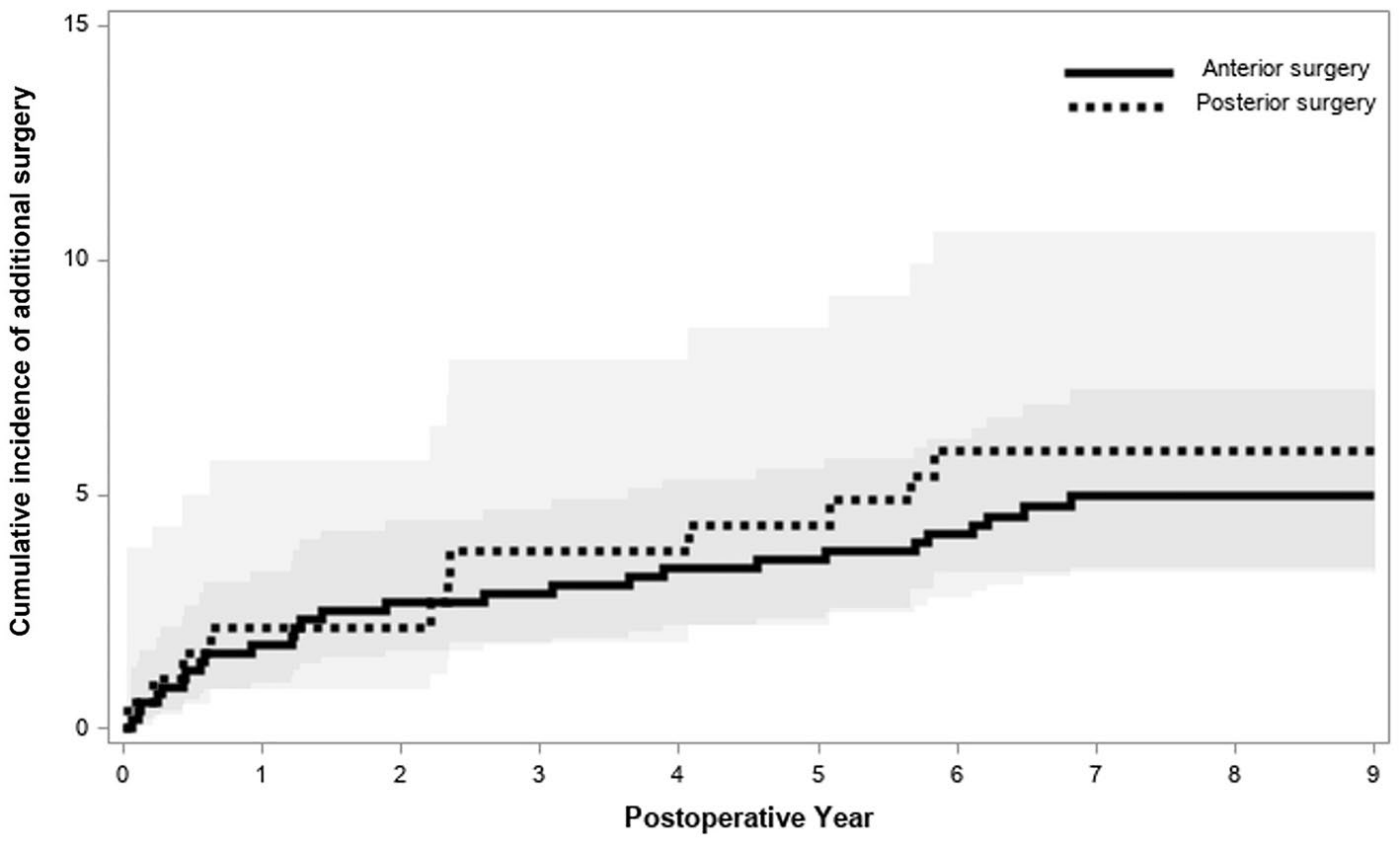
Cumulative incidence of additional surgery. The solid lines represent anterior cervical surgery and the dotted line represents posterior cervical surgery. Shaded region indicates a 95% confidence interval (CI). During the follow-up period for a median of 7.3 years, additional surgery was performed for 5.0% of patients after anterior cervical surgery and 6.5% of patients after posterior cervical surgery. The adjusted additional surgery rate after anterior cervical surgery was not significantly lower than that for posterior cervical surgery (adjusted subhazard ratio, 0.83; 95% CI 0.40–1.74).

The unadjusted SHR (0.74; 95% CI 0.38–1.47) of additional surgery was not markedly different between the anterior and posterior cervical surgeries. Further analysis, examining the hazard ratios for four specific posterior procedures (decompression and fusion, laminectomy, laminoplasty, and discectomy) and two specific anterior procedures (discectomy and corpectomy and fusion) also revealed no statistically significant difference in the additional surgery rate according to the type of surgery (Table 2). A subgroup analysis regarding age showed that the additional surgery rate for anterior surgery was low in non-elderly (age under 70 years old) and estimated to be higher in elderly (aged 70 years and older), but this difference was not statistically significant (*p* = 0.1364). After adjusting for covariates, the SHR (0.83; 95% CI 0.40–1.74) of additional surgery after anterior cervical surgery was not significantly lower than that after posterior surgery (Table 2, Fig. 2). For additional surgery, anterior surgery was chosen as a surgical method in approximately 50% of the patients. The detailed surgical methods used for additional surgery are presented in Table 3.

**Table 2:** Risk factors for additional surgery.

	Censor	Event	Unadjusted SHR (95% CI)	*P* value	Adjusted SHR (95% CI)	*P* value
Surgical method, n (%)						
Posterior cervical surgery	155	12	1		1	
Anterior cervical surgery	494	28	0.74 (0.38–1.47)	0.39	0.83 (0.40–1.74)	0.62
Posterior cervical surgery						
Decompression and fusion	12	2	1.72 (0.16–19.02)	0.66	2.04 (0.18–23.22)	0.57
Laminectomy	36	1	0.43 (0.03–7.25)	0.56	0.35 (0.02–6.19)	0.47
Laminoplasty	15	3	2.64 (0.27–26.19)	0.41	2.50 (0.25–24.51)	0.43
Discectomy	92	6	1.09 (0.13–9.46)	0.94	1.31 (0.11–15.19)	0.83
Anterior cervical surgery						
ACCF	14	1	1		1	
ACDF	480	27	0.88 (0.11-6.76)	0.90	1.03 (0.11–9.29)	0.98
Disease, n (%)						
Central stenosis	132	11	1		1	
Foraminal stenosis/disc herniation	517	29	0.75 (0.38–1.48)	0.40	0.89 (0.43–1.86)	0.76
Sex, n (%)						
Male	406	29	1		1	
Female	243	11	0.66 (0.33–1.31)	0.23	0.66 (0.31–1.44)	0.30
Age, years (%)						
Continuous			1.01 (0.99–1.04)	0.37	1.00 (0.97–1.04)	0.86
18–39	80	5	1			
40–59	427	23	0.83 (0.32–2.15)	0.70		
60–69	117	8	0.90 (0.29–2.74)	0.85		
70 and above	25	4	1.75 (0.48–6.41)	0.40		
Insurance, n (%)						
General	628	37	1		1	
Medicare	21	3	2.05 (0.63–6.73)	0.24	1.48 (0.42–5.23)	0.54
Disability, n (%)						
None	587	32	1		1	
Mild	53	6	2.11 (0.88–5.05)	0.09	1.88 (0.76–4.66)	0.17
Moderate	9	2	3.30 (0.77–14.07)	0.11	2.77 (0.52–14.89)	0.23
Type of hospital, n (%)						
Tertiary referred hospital	179	12	1		1	
General hospital	163	11	0.95 (0.42–2.17)	0.91	0.91 (0.40–2.09)	0.82
Hospital	284	16	0.85 (0.40–1.8)	0.67	0.93 (0.44–1.95)	0.85
Clinic	23	1	0.71 (0.09–5.65)	0.75	0.75 (0.09–6.42)	0.79
Charles comorbidity index						
0	422	25	1		1	
≥ 1	227	15	1.04 (0.55–1.97)	0.90	0.72 (0.37–1.43)	0.35
Diabetes mellitus						
No	559	30	1			
Yes	90	10	1.77 (0.86-3.64)	0.12	1.76 (0.91-3.43)	0.10
Osteoporosis						
No	537	32	1		1	
Yes	112	8	1.07 (0.51–2.24)	0.87	1.10 (0.45–2.69)	0.84

*CI* confidence interval, *SHR* subhazard ratio.

**Table 3:** Surgical method for additional surgery.

	Anterior surgery	Posterior surgery				Both
		Decompression and fusion	Laminectomy	Laminoplasty	Discectomy	
Posterior surgery (n = 12)	6	1	0	2	2	1
Anterior surgery (n = 28)	13	7	4	2	1	1

## Discussion

### Overview

The objective of the present study was to determine the additional surgery rate after anterior or posterior surgery for degenerative cervical spinal disease. Our findings revealed that anterior surgery was selected in 75% of the patients, and the proportion of anterior surgery was higher in patients with central stenosis than in those with foraminal radiculopathy. A significant difference between the rates of additional surgery after anterior and posterior cervical surgeries was not found. Although this was a retrospective cohort study, it may be helpful in deciding on the use of anterior or posterior surgery based on the overall outcomes of surgeries for degenerative cervical spinal disease and in adjusting health insurance-related policies.

### Surgical outcomes after anterior or posterior surgery

Several prospective studies have compared the outcomes of anterior and posterior cervical surgeries^3,19,22^. Ghogawala et al.^3^ performed a randomized controlled trial to compare anterior and posterior surgery for cervical spondylotic myelopathy. Although the clinical outcomes were similar between anterior and posterior surgeries, the complication rates were higher after anterior cervical surgery (48% vs 24%, *P* = 0.002). Dysphagia was the most common complication after anterior cervical surgery (41%), and new neurological deficit (9%) and readmission (7%) were common complications after posterior cervical surgery^3^. Additional surgery was performed in 6% of the patients after anterior cervical surgery and 4% of the patients after posterior cervical surgery^3^. Inose et al. compared the clinical and radiological outcomes of laminoplasty and anterior and posterior decompressions with fusion in a prospective cohort study. Clinical outcomes such as the Japanese Orthopaedic Association score, European quality of life-5 dimension, and neck disability index (NDI) were not different among the patient groups^19^. However, radiological outcomes such as cervical lordosis and C2–7 sagittal vertical axis worsened after posterior cervical surgery. Sakai et al.^22^ compared cervical sagittal alignment between anterior surgery and laminoplasty in a prospective cohort study. They showed that the recovery was similar for the surgical techniques in patients with balanced cervical alignment, but not in patients without balanced cervical alignment^22^. Seng et al.^16^ compared anterior cervical surgery and laminoplasty for multilevel cervical myelopathy and showed that the clinical outcomes were similar between them, but anterior cervical surgery involved higher complication rates and longer operation times.

Several systematic reviews have also compared anterior and posterior cervical surgeries. Yoshii et al.^5^ compared anterior cervical surgery and laminoplasty and showed that although the clinical outcomes were similar, postoperative cervical lordosis was better after anterior cervical surgery. Furthermore, Yoshii et al.^6^ compared fusion surgeries between anterior and poster approaches. They showed that the overall clinical outcomes were similar, but postoperative NDI and cervical lordosis were better after anterior cervical surgery^6^. Montano et al.^8^ compared ACDF and laminoplasty for cervical spondylotic myelopathy and found similar surgical outcomes. They reported that although cervical lordosis was better after ACDF, the cervical range of motion was better after cervical laminoplasty^8^. Xu et al. also reported that the overall clinical outcomes were similar between ACDF and laminoplasty. Despite better cervical lordosis after ACDF, the complication rates were higher after ACDF than after laminoplasty^3,9,13,21,22^. Tetreault et al.^10^ performed a systematic review of complications and found that the surgical approach did not influence the complication rate. Rather, patient factors such as age, body mass index, smoking habit, baseline severity score, and operation time were associated with the complications^10^.

Liu et al.^14^ compared the clinical outcomes and complication rates between anterior and posterior cervical surgeries with respect to surgical levels. If the surgical levels were less than three, clinical outcomes were superior in anterior cervical surgery; however, the complication rates were similar. Contrarily, if the surgical levels were more than three, the complication rates were higher in the anterior cervical surgery group, with similar clinical outcomes. In addition, operation time and blood loss were significantly higher after the anterior cervical surgery^14^. Based on those results, the authors recommended laminoplasty for surgical levels of more than three^14^.

Surgical decisions were made after considering the results. The present study used additional surgery as a surrogate to evaluate crude surgical outcomes. The rate of additional surgery was neither different between the surgical techniques nor was it higher than that shown in the previous studies^3^. This implies that the overall quality of the surgical treatments seemed to be well managed.

### Selection between anterior and posterior cervical surgeries

In cervical spinal surgery, the choice of the surgical technique is not a simple decision. Patient and surgeon factors as well as research outcomes influence such decisions. Additionally, one surgical technique does not fit every case; hence, there should be a trade-off between the positive and negative factors for each surgical technique. Therefore, the surgical decision in cervical spinal surgery is not unanimous and individualized on a case-by-case basis^4,25–27,46–49^. Nonetheless, a consensus is reached through continued education with research, academic meetings, and correspondence between surgeons.

Veeravagu et al. analyzed nationwide registered patient data and showed that the proportions of anterior, posterior, and combined surgeries were 85%, 13.2%, and 2.7%, respectively^31^. A similar trend was observed in a prospective observational multicenter cohort study by Fehlings et al., who compared anterior and posterior cervical surgeries. The surgical technique was at the discretion of the surgeons, and anterior cervical surgery comprised 64% of all surgeries^50^. The clinical outcomes were not significantly different between the surgical techniques. They demonstrated for the first time that when patient and disease factors are controlled, anterior and posterior cervical surgical techniques have equivalent efficacies in the treatment of cervical spondylotic myelopathy^50^.

Ossification of the posterior longitudinal ligament (OPLL) in the cervical spine is more prevalent in East Asian countries including the Republic of Korea (ROK), than in other countries^51^. In determining the surgical approach, the presence and characteristics of OPLL are critical factors. These characteristics include the level of the spine involved, the presence of the double layer sign, the type of OPLL, and the morphology of OPLL, such as the beak type^18,52,53^. Unfortunately, this information was not available in the NHIS database and as a result, we had to exclude OPLL from this study, despite its relative prevalence and importance in cervical spine surgery decision-making.

The findings of the present study revealed that anterior surgery was more frequently performed, particularly in patients with central stenosis. This trend is common among spinal surgeons. The surgical outcomes were optimized when the surgical techniques were adopted based on recommendations from established reports.

### Variations in surgery ratios across different countries and databases

Studies conducted in other countries using nationwide data have reported varying ratios of anterior to posterior surgeries. Liu et al.^54^ analyzed the national inpatient sample (NIS) database and found that from 2001 to 2013, 56.21 (79.95%) of surgeries were anterior and 14.12 (24.61%) were posterior. Virk et al.^55^ compared the anterior and posterior approach using the Medicare 5% National Sample Administrative Database (SAF5) and the Humana orthopedic database (HORTHO) of private payers from 2005 to 2014, finding 3851 (84.5%) anterior surgeries and 705 (15.5%) posterior surgeries. Veeravagu et al.^1^ used MarketScan data for the years 2006–2010 and found that 30,600 (87.4%) surgeries were anterior and 4405 (12.6%) were posterior. The variations in proportions may be attributed to various elements, including the country, time period, database characteristics, data collection methods, and preferences of surgeons. Given that a reasonably controlled degree of bias has been achieved in each sample and data collection method, these results offer valuable insights into the variations in surgical practices across different countries. However, it's important to keep in mind that these findings should not be considered a comprehensive representation of the entire nation.

### Recent advancements and changes in cervical spine surgery

Recent advancements in surgical experience, knowledge, instruments, and techniques have led to significant changes in surgical trends, especially in the field of Minimally Invasive Spine Surgery (MISS) for the cervical spine, including endoscopic surgery, navigation-guided percutaneous screw fixation, and robot-assisted procedures^56–58^. However, our study was designed to evaluate the long-term follow-up results of surgeries performed between 2006 and 2009 and thus does not reflect these recent changes. It is important to note that the results of our study, which focused on surgeries performed over a decade ago, should not be considered a representation of current surgical trends in the cervical spine.

### Analysis of nationwide data

Nationwide data are useful for investigating the overall outcomes. However, the secondary data lacked clinical and radiological information; hence, a detailed investigation and interpretation was not possible. Usually, reoperation and complication rates as well as incurred costs were used as a indicator for outcomes^32,33,37,46,59^. Morishita et al.^2^ analyzed nationwide inpatient data in Japan to compare anterior and posterior decompression and fusion. They showed that systemic complications such as respiratory failure, pneumonia, and dysphagia occurred more frequently after anterior cervical surgery, while posterior cervical surgery was more expensive^2^. Veeravagu et al. analyzed complications and cost of surgery. The overall complication rates were 15.6% after ACDF, 29.2% after posterior fusion, 22.4% after laminoplasty, and 41.1% after combined anterior and posterior surgery^31^. Although the cost of surgery was similar between anterior and posterior cervical surgeries, the high complication rate was a significant drive for higher overall cost of anterior cervical surgery^31^. Wadhwa et al., in their nationwide data analysis, also showed that reoperation rates were similar between ACDF and laminoplasty. Although the complication rate was lower after ACDF, its cost was higher than that of laminoplasty^1^. Focusing on the statistical pitfalls, although the present study did not show a significant difference in the rates of additional surgery after posterior and anterior cervical surgeries (adjusted SHR, 0.83; 95% CI, 0.40–1.74), it may be due to a type II error. Under the assumption that SHR is 1.5^3^ and cumulative incidence at 8 years for the competing event are 0.06 for anterior and 0.12 for posterior cervical surgeries based on the present data, a two-sided Gray test with the overall sample size of 711 subjects and a median follow-up time of 7.5 years achieves approximately 49% power at a 0.05 significance level.

### Limitations

The present study showed the nationwide rate of additional surgery using national health insurance data, which may be useful in overseeing the current status and adjusting the national health insurance policy. However, this study had some limitations.

### Characteristics of database

First, the sample size was relatively small because this study used sample data. Moreover, the patients were relatively younger than the published age^31^. There was a chance of selection bias when using sample data. Therefore, this study has the chance of a type II error as discussed before^3^. We acknowledge the limitations and recommend using data of all patients to study cervical spinal surgery. Second, there was a possibility of errors or missing information in the input of disease or procedure codes. The NHIS database is based on data collected from physicians for medical fee claims, which inherently poses a risk of errors or missing data. This is another potential source of bias, given the limitation of using claim data. Given the potential for bias, the results of this study should not be considered as fully representative of the nation and should not be directly used in the clinical decision-making process. Third, as the National Health Insurance Service (NHIS) data is secondary data and does not include all clinical or radiological data, such as surgical site infection, postoperative complications, location and the number of surgeries, cervical kyphosis, and other radiological parameters. We could only utilize the available basic information such as types of diseases, gender, age, types of insurance, disability grade, types of hospitals, the Charlson comorbidity index, diabetes mellitus, and osteoporosis. Some of these are known risk factors for revision surgery, but a thorough comparison of surgical outcomes with all clinical and radiological information was not possible. Finally, our study data focused on surgery from 2006 to 2009 to find out the long-term follow-up results. However, since our focus was on a period more than a decade ago, we should not interpret the results as reflecting current trends.

### Considerations in clinical practice

First, the selection of surgical techniques was not standardized. In the ROK, all doctors must fulfill at least 24 h of education credit every 3 years to maintain a doctor’s license. In addition, the NHIS does not limit the freedom of patients to visit any hospital or doctor in any area as many times as they want. Therefore, surgeons should update their knowledge and surgical skills to keep up with other doctors and hospitals. Nonetheless, the surgical techniques and outcomes were not the same among surgeons across the country. Second, the selection of anterior or posterior cervical surgery has yet to be standardized among all surgeons. We assumed that all surgeons followed the general guidelines of evidence-based medicine; however, this was not verified, and the odds of selecting a surgical technique were not the same for every patient. Third, the decision and surgical skills were not uniform among surgeons. Numerous factors, including clinical and radiological parameters, should be considered when deciding the surgical technique. In addition, the number of surgical levels also influenced the outcome; however, it was not controlled in this study because the surgical extent was not recorded in the database. Moreover, the current surgical techniques, philosophy, and indications differ from the time of this study. Finally, the surgical level was not registered in the database, and additional surgery included operations at both the index and other cervical levels. Consequently, the rate of additional surgery in this study should not be interpreted as failure at the index level. Therefore, the present results cannot be referenced for clinical purposes because of these limitations. Nonetheless, the purpose of this study was to examine the overall picture of actual clinical practice, and this study may be meaningful in this regard.

## Conclusion

The rates of additional surgery were not different between anterior and posterior cervical surgeries for degenerative cervical spinal disease in an analysis of a nationwide sample in the ROK. Although it may not be directly applicable to clinical practice, it would be helpful to evaluate the current practice and adjust health-related policies.

## Data Availability

The data analyzed in this study belongs to the NHIS, and access to the raw data is only available through a secure remote connection to the NHIS database server. In accordance with the laws of Korea, copying, transporting or sharing the raw data is strictly prohibited. Therefore, the raw data could not be included as supplementary material in this publication, and we could only include the statistically processed data, figures, and tables that were obtained through the remote connection during the course of the study. The NHIS-NSC data is available to researchers who are interested by following the appropriate request and review process outlined on the NHIS website (https://nhiss.nhis.or.kr/bd/ab/bdaba021eng.do).
